# Fructose contributes to the Warburg effect for cancer growth

**DOI:** 10.1186/s40170-020-00222-9

**Published:** 2020-07-10

**Authors:** Takahiko Nakagawa, Miguel A. Lanaspa, Inigo San Millan, Mehdi Fini, Christopher J. Rivard, Laura G. Sanchez-Lozada, Ana Andres-Hernando, Dean R. Tolan, Richard J. Johnson

**Affiliations:** 1 Department of Nephrology, Rakuwakai Otowa Hospital, 2 Otowa-Chinji-cho, Yamashina-ku, Kyoto, Japan; 2 Department of Stem Cell Biology & Regenerative Medicine, Shiga University of Medical Science, Otsu, Japan; 3 Division of Renal Diseases and Hypertension, University of Colorado Denver, Aurora, CO USA; 4 Department of Medicine, Division of Endocrinology, Metabolism and Diabetes, University of Colorado School of Medicine, Aurora, USA; 5 University of Colorado Cancer Center, Aurora, CO USA; 6 Department of Medical Oncology, University of Colorado Denver, Aurora, CO USA; 7 Department of Cardio-Renal Physiopathology, Instituto Nacional de Cardiología Ignacio Chavez, 14080 Mexico City, CP Mexico; 8 Department of Biology, Boston University, Boston, MA USA

**Keywords:** Fructose, Uric acid, Cancer, Hypoxia, Mitochondria, Lactate, Polyol pathway

## Abstract

Obesity and metabolic syndrome are strongly associated with cancer, and these disorders may share a common mechanism. Recently, fructose has emerged as a driving force to develop obesity and metabolic syndrome. Thus, we assume that fructose may be the mechanism to explain why obesity and metabolic syndrome are linked with cancer. Clinical and experimental evidence showed that fructose intake was associated with cancer growth and that fructose transporters are upregulated in various malignant tumors. Interestingly, fructose metabolism can be driven under low oxygen conditions, accelerates glucose utilization, and exhibits distinct effects as compared to glucose, including production of uric acid and lactate as major byproducts. Fructose promotes the Warburg effect to preferentially downregulate mitochondrial respiration and increases aerobic glycolysis that may aid metastases that initially have low oxygen supply. In the process, uric acid may facilitate carcinogenesis by inhibiting the TCA cycle, stimulating cell proliferation by mitochondrial ROS, and blocking fatty acid oxidation. Lactate may also contribute to cancer growth by suppressing fat oxidation and inducing oncogene expression. The ability of fructose metabolism to directly stimulate the glycolytic pathway may have been protective for animals living with limited access to oxygen, but may be deleterious toward stimulating cancer growth and metastasis for humans in modern society. Blocking fructose metabolism may be a novel approach for the prevention and treatment of cancer.

## Introduction

Obesity and metabolic syndrome are strongly associated with some types of cancer, but it remains unknown if there is a common mechanism. In 1924, Otto Warburg initially described that cancer cells, as opposed to normal cells, exhibit a unique property to ferment glucose into lactate even in the presence of sufficient oxygen [1, 2]. This glycolytic pathway has been thought to be a key energy source and is now called the “Warburg effect.” Understanding the glycolytic pathway may provide insights into the mechanism that links metabolic syndrome and cancer.

Glucose is a key glycolytic substrate for cancer and serves not only for an energy source, but also for the anabolic production of metabolites including serine, aspartate, nucleotides, and fatty acids, and for redox regulation [3–6]. An enhanced glucose metabolism in cancer can be monitored by positron emission tomography (PET) with enhanced cellular uptake of [^18^F]-FDG (2-deoxy-2-[^18^F]-fluoro-d-glucose). However, FDG-PET imaging often fails to detect some types of cancers. Lassen et al. showed that PET could successfully detect only 45% of the primary tumors in patients with a variety of metastases [7]. One potential explanation is that glucose is not a common energy source for all types of tumors as one of the major glucose transporters, GLUT1, was detected in only 87 out of 154 human malignant tumors [8]. In addition, Guppy et al. demonstrated that the contribution of glucose with or without glutamine to total ATP turnover was 40% or 28%, respectively, in MCF-7 breast cancer cell line [9]. These data suggest that there might be other sources of energy for cancer growth.

Recently, fructose has emerged as a key driving force in the recent epidemic of metabolic syndrome. Interestingly, fructose is also capable of inducing mitochondrial dysfunction and producing oxidative stress, which in turn suppresses aconitase in TCA cycle. As a result, fructose metabolism preferentially downregulates mitochondrial function and preferentially stimulates the glycolysis pathway [10]. Given these facts, fructose might be an alternative energy source for cancers. Here we discuss the role of fructose as a potential preferred substrate for cancer growth and metastases.

## Role of fructose under physiological and pathological condition

Fructose is a simple sugar present in fruit (fruit sugar), which has an identical chemical composition with glucose (C_6_H_12_O_6_). Fructose can be converted into glucose under certain conditions (gluconeogenesis) whereas glucose can also be converted to fructose by the polyol pathway [11]. In the kidney, several precursors are a substrate for gluconeogenesis, but fructose is preferentially utilized and physiologically converted into glucose in the renal proximal tubular cells. The proximal tubular cells express GLUT5 and fructokinase exclusively with a series of enzymes for gluconeogenesis, but not for glycolysis [10]. Glucose produced in the proximal tubules is released into systemic circulation in order to maintain serum glucose concentration at physiological levels, in particular during starvation or while fasting during sleep [12, 13].

In turn, the placenta enzymatically metabolizes glucose into fructose during pregnancy in various species including ungulates, cetaceans, and humans [14–16]. Importantly, fructose is timely produced at the early phase of pregnancy when fetal organ growth is processed under a low oxygen condition [17, 18]. Investigating the role of fructose, White et al. [19] injected [U-^14^C]-fructose into fetal pig, examined the effect in several organs, and found ^14^C was incorporated into nucleic acids, especially in the RNA, in the skeletal muscle and liver, and was significantly greater than incorporation in the DNA in skeletal muscle. Therefore, fructose metabolism likely contributes to the fetal organ development by stimulating synthesis of nucleic acid, lipid, NAPDH, and hexosamine [20, 21].

Epidemiologic, experimental, and clinical studies suggest that intake of sugar and HFCS could be a cause for the current epidemic of metabolic syndrome and obesity [22]. The detrimental effects of fructose have been confirmed by recent studies showing that a low fructose diet could provide several benefits for health, such as lowering blood pressure and reducing inflammatory factors, including C-reactive protein and soluble intracellular adhesion molecule-1 [23–26]. In particular, Schwimmer et al. recently performed a randomized clinical trial with 40 children with active non-alcoholic fatty liver disease (NAFLD) and examined the effect of low sugar diet for 8 weeks. It was found that hepatic fat accumulation was significantly improved by low sugar diet compared to normal diet [26]. Other studies have also shown a benefit from isocaloric restriction of fructose on various features including fatty liver, hypertriglyceridemia, and insulin resistance [25, 27, 28].

**Fig. 1:**
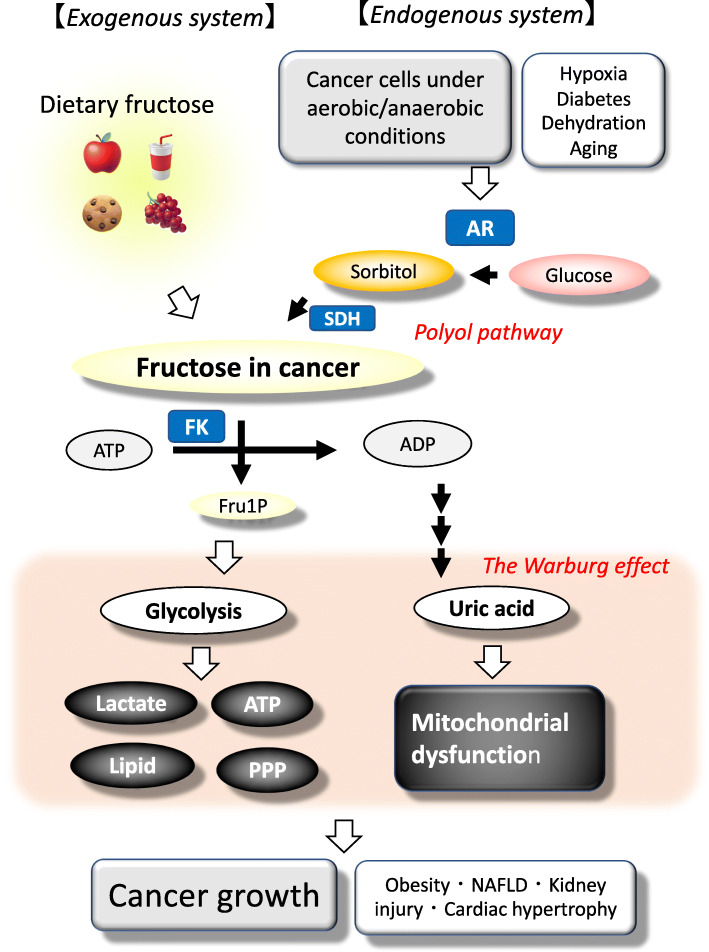
The conceptual schema of our hypothesis for the role of exogenous vs. endogenous fructose for the Warburg effect and cancer growth. AR, aldose reductase; FK, fructokinase; SDH, sorbitol dehydrogenase; PPP, pentose phosphate pathway; NAFLD, non-alcoholic fatty liver disease

Several groups, including ours, have proposed that the recent increase in dietary fructose consumption contributes to the epidemic of obesity and metabolic syndrome [29, 30]. However, fructose can also be endogenously produced in several pathological conditions, including diabetes, ischemic cardiac and kidney injury, and salt-induced metabolic syndrome [11, 31–34]. A potential mechanism is that high glucose, ischemia, and high osmolarity activate the polyol pathway, in which glucose is sequentially converted to sorbitol by aldose reductase, and then oxidized to fructose by sorbitol dehydrogenase. Recently, Mirtschink et al. showed that the cardiac myocytes were capable of producing fructose endogenously, and the fructose generated was involved in the pathological process of cardiac remodeling. Specifically, fructokinase was identified as a HIF-1α-mediated factor which was induced in the hypertrophic heart model induced by hypertension in either the 1-kidney-1-clip (1K1C) model or by transverse aortic constriction (TAC) [34]. They also reported that there was upregulation of fructokinase in cardiomyocytes obtained from biopsies of patients with hypertrophic cardiomyopathy. A pathological role of endogenous fructose was also demonstrated in models of diabetic nephropathy, acute tubular injury, metabolic syndrome, and cardiac hypertrophy [11, 31–33] (Fig. 1). A summary of mouse models in which endogenous fructose has been shown to play a pathogenic role is shown in Table 1.

**Table 1:** Endogenous fructose contributes to several types of disease progression

Organ	Type of disease	Ref.
Kidney	Renal tubular injury in diabetic mice	[11]
	Ischemia-induced renal tubular injury in mice	[31]
	Aging kidney in mice	[35]
	Dehydration-associated kidney injury in mice	[36]
Heart	Hypertension-associated cardiac hypertrophy in mice	[34]
Systemic	High salt-induced metabolic syndrome in mice	[33]

## Consequence of fructose metabolism

Fructose is firstly metabolized by fructokinase (known as ketohexokinase), which phosphorylates fructose to produce Fructose 1-phosphate (Fru1P). It was found that fructokinase is expressed most abundantly in the liver, so that the liver was originally thought to be the primary site for dietary fructose metabolism [37, 38]. However, Jang et al. [39] demonstrated that dietary fructose is primarily cleared by the intestine while higher doses overcome the intestinal fructokinase capacity and reach the liver and circulation. Likewise, Zhao et al. showed using mice that dietary fructose is converted to acetate by the gut microbiota [40]. These data suggest that gastrointestinal tract plays a substantial role in fructose metabolism. However, recent experiments using mice with the selective knockout of fructokinase in the liver or intestine document that, while the intestine has an important role in clearance and intake, the liver metabolism of fructose is responsible for most of the features of metabolic syndrome [41].

**Fig. 2:**
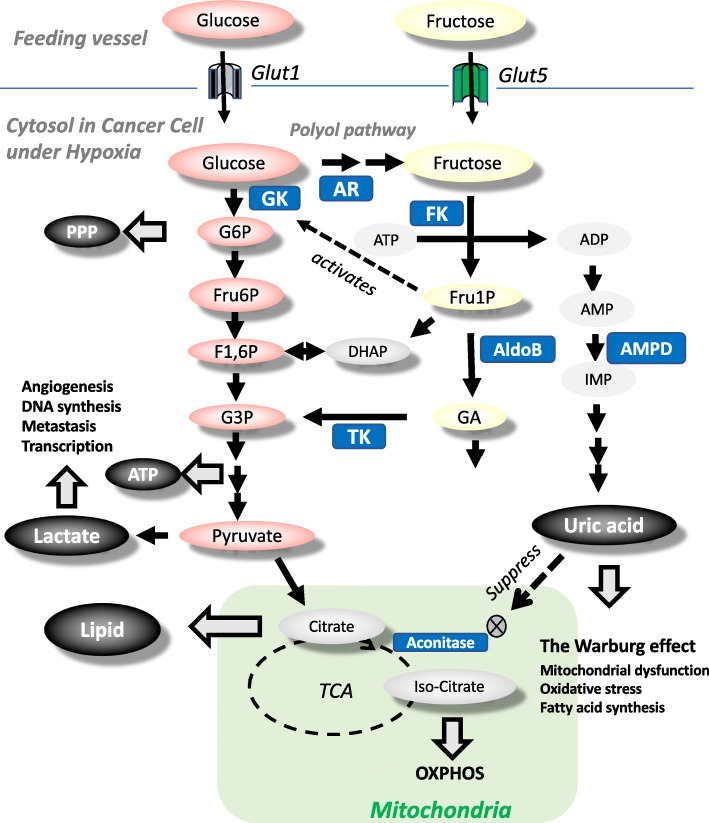
Glucose and fructose metabolism for cancer growth. Uric acid blocks aconitase, resulting in the disconnection of fructose metabolism from mitochondrial respiration. Uric acid is a byproduct of fructose metabolism and inhibits aconitase. As a result, fructose metabolism is disconnected from mitochondrial oxidative phosphorylation (OXPHOS), but maintains other metabolic pathways for pentose phosphate pathway (PPP), lactose production, ATP production, and lipid synthesis, all of which likely contributes to the cancer growth. Fructose 1 phosphate (Fru1P) competitively activates GK by releasing from glucokinase regulatory protein (GKRP), accounting for fructose facilitation of glucose utilization. AR, aldose reductase; FK, fructokinase; AldoB, aldolase B; AMPD, AMP deaminase; TK, triokinase

Fru1P is subsequently metabolized by aldolase B and triokinase to dihydroxyacetone phosphate and glyceraldehyde-3-phosphate to enter the glycolytic pathway distal to phosphofructokinase. Recently, a key role of aldolase B in cancer growth was shown by Bu et al. using mouse models that aldolase B mediates colon cancer liver metastasis and that reducing dietary fructose diminishes liver metastatic growth [42]. The initial steps of fructose metabolism activates the aerobic glycolysis pathway to generate ATP and to turn on the pathological activation of gluconeogenesis and lipogenesis, and finally glucose, glycogen, triglycerides, and lactate are produced (Fig. 2). Fructose acts as a carbon source and stimulates some intracellular signaling, including carbohydrate-responsive element-binding protein (ChREBP) [43, 44] and glucokinase regulatory protein (GKRP) [45, 46]. In parallel, fructokinase activation sequesters a phosphate, so that intracellular phosphate and ATP levels are transiently reduced [47]. The rapid reduction of phosphate consequently activates AMP deaminase, which cleaves AMP to IMP. However, the phosphate levels subsequently increase due to the slower aldolase reaction with Fru1P. This reaction is further accentuated by the increased IMP, which is an aldolase B inhibitor [48]. This overall events drive uric acid production [22, 44, 49]. In turn, a recent study using a mouse model demonstrated that fructose-mediated fatty liver disease is likely mediated by impairment of fatty acid oxidation due to deacetylation of Acyl-CoA dehydrogenase, long chain (ACADL) and carnitine palmitoyl- transferase 1α (CPT1α) [50].

## Clinical associations of fructose intake with cancer

The idea that cancer cells might utilize fructose as a fuel is supported by the observation that GLUT5, the primary fructose transporter, is expressed on the cell surface of several types of tumors. In the 1990s, several research groups found that GLUT5 was expressed in human epithelial colorectal adenocarcinoma cells as well as human breast cancer cells [51–53]. Subsequently, a cohort study was conducted to evaluate the association of fructose with pancreatic cancer. In a study involving 88,802 women in the Nurses’ Health Study, fructose intake was found to be the strongest risk factor for pancreatic cancer in subjects who were overweight or sedentary [54]. Three years later, another study that combined the Nurses’ Health Study and the Health Professionals Follow-up Study showed that sugar-sweetened beverage consumption was associated with an increase in risk for pancreatic cancer among women, but not men [55]. Another prospective study using a food-frequency questionnaire in which 77,797 women and men were followed for a mean of 7.2 years in Sweden also found that high consumption of sugar and high-sugar foods resulted in a greater risk of pancreatic cancer [56]. These data suggested that dietary fructose could be a risk for pancreatic cancer, and this notion was later supported by the finding that serum concentration of fructose was also higher in patients with pancreatic cancer than healthy patients [57].

Likewise, there is a positive association between sugar or fructose intake and colorectal cancer. Many studies found a positive association between sugar/fructose intake and the risk of colorectal cancer, but other studies were negative. For example, Michaud et al. examined 1809 subjects with two prospective cohort studies, the Nurses’ Health Study and the Health Professionals Follow-up Study, to show that a small increase in risk was observed in men with increased consumption of sucrose or fructose, and this association was stronger among men with elevated body mass index [58]. In contrast, Terry et al. analyzed the data from a cohort of 49,124 women participating in a randomized controlled trial of screening for breast cancer in Canada and showed that total sugar intake did not predict colorectal cancer risk [59].

**Fig. 3:**
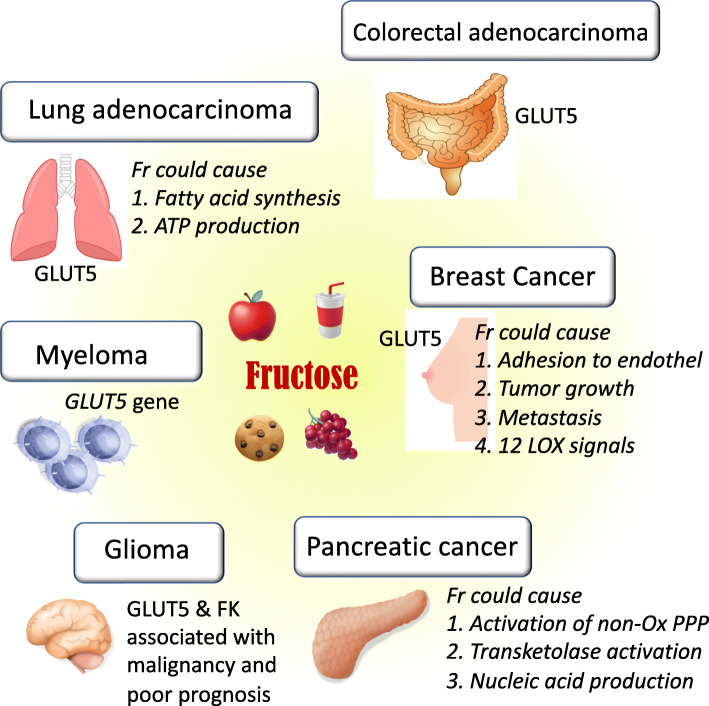
Several types of human cancers, which would utilize fructose as a fuel energy. Clinical studies show that either GLUT5 protein or *GLUT5* gene is expressed in lung adenocarcinoma, colorectal adenocarcinoma, breast cancer and myeloma. Effects of fructose in the human cancer cell line are shown by *Italic*. The separated part indicates mouse study showing that dietary fructose could mediate intestinal cancer by activation of fructokinase and lactate production. LD, non-alcoholic fatty liver disease

Other types of cancer could be also mediated by fructose. For example, an increase in GLUT5 expression was associated with poor prognosis in patients with lung adenocarcinoma [60]. Likewise, Chen et al. also showed that AML patients exhibited upregulated expression of *GLUT5* gene on myeloid cells, while increased fructose utilization was associated with poor clinical outcomes [61]. In brain, it was also found that fructokinase and GLUT5 were highly expressed in glioma and were also correlated with malignancy and poor survival of glioma patients [62, 63]. Summary is shown in Fig. 3 and Table 2.

**Table 2:** Fructose effects in various types of cancer cells

Types	Fructose effects	Material	Ref.
Pancreatic cancer	Activation of non-oxidative PPP Transketolase activation Nucleic acid production	Cultured cell line (CaPan-I, CaPan II, HPAF2, Aspc1, Panc-1, MiaPaCa-2)	[64]
Lung cancer	Fatty acid synthesis ATP production	Human bronchial epithelial cell (BEAS-2B); NSCLC cells (PC-9, H1299, A549, HCC-827, H1975)	[60]
Breast cancer	Adhesion to endothelium Tumor growth Metastasis 12 LOX signals	Cultured cell (MDA-MB-468 cell, MCF-7 cell) Mice (FVB/N-Tg(MMTVneu)202Mul/J)	[8, 9, 65, 66]
Intestinal cancer	Fructokinase Lactate production	Mice	[67]

## Fructose plays a distinct role from glucose in cancer growth

If fructose is utilized as a fuel for several types of cancer, there may be a distinct advantage of fructose over glucose. This issue was examined by several investigators using cultured cancer cells lines [68]. Liu et al. found that using pancreatic cancer cells, fructose and glucose exhibited the same effect on cell proliferation, but their intracellular metabolism was different. The productions of lactate, CO_2_, and fatty acid were significantly higher in cells with glucose stimulation compared to those with fructose stimulation. In turn, fructose was more potent to stimulate the non-oxidative pentose phosphate shunt in association with intracellular transketolase activation, ribose synthesis, and uric acid production whereas glucose activated glucose-6-phosphate dehydrogenase (G6PDH) in the oxidative pentose phosphate pathway [64]. For lung cancers, Weng et al. showed that compared to glucose, fructose was more potent to produce ATP and fatty acids [60]. Interestingly, pancreatic cancer cells predominantly utilized glucose for fatty acid synthesis, thereby the potential mechanism for fatty acid synthesis seems to be distinct between lung cancer cells and pancreatic cancer cells [60, 64]. In breast cancer cells, fructose, when compared to glucose, caused greater adhesion to endothelial cells and enhanced more aggressive migration [65]. Finally, Jiang et al. performed an experimental study in mice induced with breast cancer and found that a fructose diet was more effective at stimulating tumor growth and the spread of metastatic tumors in the lung, compared to either a glucose or control starch diet. In mice, fructose also stimulated the expression of 12-lipoxygenase (12-LOX) and the production of the arachidonate metabolite 12-hydoroxy-5Z,8Z,10E,14Z-eicosate-traenoic acid (12-HETE) production, thereby implicating fructose in inducing 12-LOX signaling to increase the risk of breast cancer development and its metastasis [66]. These results are summarized in Table 1.

In turn, hepatocellular carcinoma (HCC) appears distinct from other cancers as fructose metabolism is reduced in HCC compared to healthy hepatocytes [69]. Fructokinase is known as ketohexokinase (KHK) and has two isoforms: KHK-C and KHK-A. KHK-C has a greater affinity and a lower *Km* value for fructose compared to KHK-A. KHK-C rapidly metabolizes fructose to Fru1P and is considered to be the primary enzyme for fructose metabolism [38]. In contrast, KHK-A is expressed at low levels in a wide range of tissues, and the precise role of KHK-A remains to be determined. However, an experiment using the KHK-A-specific knockout mouse indicates that KHK-A might reduce fructose metabolism in the liver and prevent the development of metabolic syndrome [38]. Alternatively, KHK-A expressing in other tissues might play a role in metabolism of fructose that overflows the intestine. Recently, Li et al. showed that HCC cells have reduced fructose metabolism by switching from high-activity fructokinase (KHK-C) to the low-activity KHK-A isoform. In HCC, KHK-A acts as a protein kinase, phosphorylating phosphoribosyl pyrophosphate synthase to promote PPP-dependent nucleic acid synthesis and HCC development [69].

The role of hexokinase in phosphorylating fructose to fructose 6-phosphate (Fru6P) (this directly entering glycolysis) in cancer cells remains unclear. While fructose is preferentially metabolized by KHK-C in several organs, it has been shown that hexokinase plays a significant role in fructose metabolism, nearly as much as that of KHK, in mouse brain slices [70].

## Physiological dose of fructose could be enough to promote cancer growth

The increase in high fructose corn syrup (HFCS) consumption since 1970s was found to be associated with the epidemic of cardiovascular and metabolic diseases, indicating that fructose might play a causal role. However, clinical trials usually use higher amounts than commonly ingested in daily life, raising a question of whether such findings are clinically relevant [71]. In fact, Choo and Sievenpiper found that the average dose of fructose was 101.7 g/day in substitution trials and 187.3 g/day in addition trials compared with a mean of 49 g/day in the NHANES general population survey (1977–2004) [72]. As such, it is important to examine the effect of fructose on cancer growth at moderate concentrations that are attainable with the current Western diet. Goncalves et al. examined if the fructose amount in a typical 12 ounce sugar-sweetened beverage could contribute to the growth of intestinal cancer in mice [67]. They found that even modest amounts of fructose (~ 3% of total daily caloric intake) caused tumor growth associated with lactate production, phosphofructokinase activation, and GLUT5 induction. Importantly, knocking down fructokinase (ketohexokinase), the first enzyme involved in fructose metabolism, was found to suppress cancer growth in response to HFCS [67]. Likewise, Bu et al. examined the importance of fructose on colon cancer liver metastasis and found that reducing dietary fructose was as potent as targeting AldoB to reduce liver metastases. Interestingly, however, reducing dietary fructose had little effect on the primary tumor [42].

## Fructose facilitates glucose utilization

An additional point to be aware of is the fact that we rarely consume fructose in isolation, but together with glucose in foods and beverages using sugars, sucrose, and HFCS. Since serum glucose concentration is also constantly maintained at physiological levels, most cells are constantly supplied with a substantial amount of glucose. When a large amount of dietary fructose is consumed, serum fructose levels are raised simultaneously with glucose. Thus, the effect of fructose should be generally considered together with glucose in order to understand the pathophysiological basis. The combination of fructose with glucose influences glucokinase, the first enzyme for glycolysis. Van Schaftingen et al. [73] and Agius and Peak [74] demonstrated that glucokinase was positively regulated by Fru1P whereas it was inhibited by Fru6P in the hepatocyte. The mechanism for Fru1P activating glucokinase is by promoting the release of glucokinase from GKRP, which sequesters glucokinase in the nucleus [45, 46]. Even at small concentrations, intracellular fructose is rapidly metabolized to Fru1P. Therefore, Fru1P-induced glucokinase activation could be a mechanism for why fructose facilitates glucose utilization. Consistently, Shiota et al. showed that the effect of small amounts of fructose enhanced hepatic glucose uptake in the dog [75]. Furthermore, fructose metabolism also increases fructokinase activity, which depletes intracellular ATP. Since ATP negatively regulates the glycolytic pathway by inhibiting phosphofructokinase and pyruvate kinase, the ATP depletion due to fructokinase activation would enhance glycolysis. In fact, this phenomenon has been recently demonstrated in a model of colon cancer in mice [67].

## Uric acid is a potential mechanism for fructose-induction of the Warburg effect

In many physiological and pathological conditions, fructose is efficiently metabolized under anaerobic and aerobic conditions. However, the mechanism remains unclear. Recently, our research group has attempted to clarify the role of uric acid in fructose metabolism [22, 49]. A novel finding was that uric acid could prevent fructose metabolites from channeling into mitochondrial oxidation using the human hepatocellular carcinoma cell line HepG2 [76]. A potential mechanism was the correlation of elevated uric acid to decreased aconitase activity in the mitochondria [77], which would disconnect fructose metabolites from mitochondrial oxidation. It attributes to aconitase lying at the junction of acetyl-CoA oxidation or acetyl-CoA shuttling out of the mitochondria for fatty-acid synthesis. Consistent with this, the decreased aconitase activity from fructose-induced increased uric acid concentration leads to the accumulation of citrate, which is subsequently translocated from mitochondria to cytosol, where citrate was utilized for lipid synthesis by sequential ATP-citrate lyase and fatty-acid synthase [76]. This is shown in Figs. 1 and 2.

Recently, several investigators have re-evaluated the role of mitochondria and showed that mitochondria is commonly required for tumor growth. Weinberg et al. indicated that tumor cells would require mitochondria-derived reactive oxygen species (ROS), but not OXPHOS, for cell proliferation [78]. We previously showed that both fructose and uric acid stimulate mitochondrial ROS production with mitochondrial morphological changes in HepG2 cells while the TCA cycle is suppressed by the inhibition of aconitase [74, 76]. Therefore, uric acid likely contributes to cancer growth by generating mitochondrial ROS in spite of blocking TCA cycle.

## Lactate could contribute to cancer growth

Lactate, an end-product of cytosolic fructose metabolism, may contribute to carcinogenesis. Otto Warburg first identified the role of lactate in cancer by showing that arterial glucose uptake in tumor cells was about 47–70% compared to 2–18% in normal tissues, and tumor cells converted 66% of glucose to lactate [79]. It was also found that lactate levels were increased up to 40-fold in glycolytic tumors and correlated with cancer cell metastasis and poor survival [77, 80]. A potential mechanism is the ability of lactate to induce VEGF in endothelial cells, leading to angiogenesis and tumor growth [81]. In fact, blocking lactate production by blocking LDH-A with a chemical inhibitor or gene deletion ameliorated angiogenesis and inhibited cancer cell proliferation [82]. Lactate is likely required at multiple steps for carcinogenesis, including immune escape, cell migration/metastasis, and self-sufficiency [83].

Adding fructose onto glucose results in much more lactate production than glucose alone [84, 85]. The mechanism may be due to the fact that glycolysis is regulated during glucose metabolism as phosphofructokinase activity decreases if intracellular ATP falls or citrate accumulates, whereas in fructose metabolism there is no negative regulation of fructokinase [86].

## Fructose is preferentially utilized for cell survival under hypoxic condition

In 1955, Thomlinson and Gray performed histological examination with human lung cancer and found the presence of tissue necrosis relative to blood vessels, postulating that the degree of anoxia may play an important role in tumor viability, although they did not accurately measure oxygen tension of tumors [87]. In the 1990s, the situation changed with the invention of the oxygen electrode, which was a novel device allowing investigators to directly measure tissue oxygen levels in human tumors [88]. We now know that oxygen concentration in human tumors is heterogeneous with many regions at very low levels. Median oxygen pressure (pO_2_) in pancreatic cancer is 2.7 mmHg whereas it is more than 50.0 mmHg in normal pancreatic tissues [89]. Likewise, median pO_2_ in lung cancer, breast cancer, and prostate cancer is 7.5, 10.0, and 2.4 mmHg, respectively [89]. This suggests cancers have to be able to tolerate hypoxic condition to maintain viability and growth.

Recently, Park’s research group examined the unique characteristics of naked mole rats as to how these animals could survive for longer time compared to normal mice under hypoxic and anoxic conditions. The authors discovered that there was substantial endogenous production of fructose in several organs, including the kidney and liver, under hypoxic or anoxic conditions [90]. One potential mechanism could be that fructose metabolism reduces oxygen demand by reducing mitochondrial respiration via the effects of uric acid describe above. The increased glycolysis from Fru1P activation and the increased use of the PPP via transketolase activation provided the needed ATP, NADPH, and ribose for providing lipids, hexosaminoglycans, and nucleic acid for cell survival. A major issue for hypoxic conditions would be the concern that ATP derived from fructose metabolism may not be sufficient for cell survival or growth. However, Anundi’s group in 1987 found that fructose protected hepatocytes from hypoxic injury whereas glucose failed to show any protections [91]. A key finding would be that fructose metabolism did not reduce ATP concentration, but rather raised the ATP/ADP ratio with concomitant increases in lactate and pyruvate concentration in the liver. Likewise, Weng et al. showed that fructose accelerated ATP production compared to glucose even in a cancer cell line [60]. The potential mechanism for fructose-associated ATP production under hypoxia remains to be determined, and accelerated glycolysis would be responsible for the energy production. Alternatively, lactate can be a fuel as lactate can enter the mitochondria through MCT1 and then be oxidized to pyruvate via mitochondrial LDH and then to Acetyl CoA for the Krebs cycle [92]. Thereby, fructose-derived lactate (as opposed to or with glucose-derived lactate) may be also a key element for mitochondrial oxidative phosphorylation.

It is of interest that both fructose metabolism and hypoxic conditions are theoretically associated with a reduction in intracellular ATP levels, but the combination would often result in a rise in ATP production. Since fructose-induced ATP depletion is transient, the slower aldolase reaction with Fru1P and IMP would subsequently increase intracellular phosphate and increase ATP levels. In addition, during fructose metabolism, one molecule of ATP is consumed by the activation of fructokinase while the downstream reaction from fructose-1,6-bisphosphate (FBP) through pyruvate, which is the energy payoff phase in the glycolytic pathway, yields four molecules of ATP, accounting for positive ATP balance in the fructose metabolism. Alternatively, several studies with non-cancer cells indicated that FBP would be a key player to protect cells from ischemic injury. FBP has been suggested as being responsible for the reduced hypoxic injury in astrocytes in which ATP concentration was maintained [93, 94]. Potential mechanisms include (1) stimulation of carbohydrate metabolism through phosphofructokinase activation [95], (2) direct glycolytic metabolism of FBP resulting in ATP production [96], (3) prevention of oxygen-derived free radical injury, and (4) stabilizing intracellular calcium [97]. Further studies are needed to confirm which mechanisms would be relevant to cancer development and progression.

## Aldose reductase activation suggests endogenous production of fructose in cancers

While we are proposing that some cancer cells may become fructose-dependent, a key question is how cancer cells survive if fructose provided by the diet is not sufficient. Since serum fructose concentration is much lower compared to serum glucose levels [29, 98], this would be a critical issue for such types of cancers.

As mentioned above, humans and certain species of animals carry a unique system to endogenously produce fructose. Therefore, there is a possibility that certain types of cancer cells could also possess such system. The key enzyme that stimulates endogenous fructose production is aldose reductase in the polyol pathway. Given the fact that glucose is constantly supplied from the systemic circulation, the activation of aldose reductase could result in local fructose production [16].

We recently found that aldose reductase is activated in several organs under several pathological conditions, including ischemia, heart failure, and inflammation [99–102], leading to endogenous fructose production [11, 33]. Importantly, several researchers showed that aldose reductase is activated in various types of human cancers, including liver, breast, ovarian, cervical, and rectal cancers [103]. This evidence would suggest that fructose may be endogenously produced in those cancer cells where it could potentially stimulate cancer growth (Fig. 1).

## Perspective

Fructose has emerged as a key nutrient for cancer cells expressing GLUT5 and behaves differently from glucose. In case of the failure of FDG-PET imaging, PET fructose imaging may be a future alternative to detect certain types of cancers [104, 105]. Fructose metabolism provides several necessities for cancer cell growth, including nucleotides, lipids, and energy. An important issue is whether blocking fructose metabolism could be a therapeutic strategy. To treat such types of cancers, a low fructose diet would be one safe approach, but since fructose can also be generated endogenously, the most effect approach may involve blocking fructokinase. In humans, the absence of the fructokinase gene results in the condition of essential fructosuria intolerance which is a relatively asymptomatic condition [106], so selective pharmacological blockade of fructokinase may be an attractive approach. Alternatively, uric acid and/or lactate production could be targets since uric acid mediates multiple consequences of fructose metabolism, including enhancing both aldose reductase and fructokinase activation, and blocking aconitase to tease out the effect of fructolysis from mitochondrial respiration. Currently, xanthine oxidase inhibitors are commercially available and are widely used in clinical medicine, and therefore, as the first step, simple experiments applying the drug to fructose-fed mice with cancer would easily address this issue.

## Conclusions

In addition to glucose, recent studies suggest that fructose could be alternative energy source for cancer growth. Fructose can be preferentially metabolized under low oxygen condition to accelerate glucose utilization, and exhibit distinct effects, including production of uric acid and lactate as major byproducts. In particular, uric acid promotes the Warburg effect by preferentially downregulating mitochondrial respiration and increasing aerobic glycolysis that may aid metastases that initially have low oxygen supply. Blocking fructose metabolism may be a novel approach for the prevention and treatment of cancer.

## Data Availability

Not applicable
